# Clinical outcomes of modified intradermal vs. traditional intermittent suture technique in total knee arthroplasty: a single-center retrospective study

**DOI:** 10.3389/fsurg.2025.1655296

**Published:** 2025-11-07

**Authors:** Changzhi Huang, Nanyi Xu, Shimin Zhang, Jiuzao Lin, Xiaoyong Wang

**Affiliations:** 1 Ningde Clinical Medical College of Fujian Medical University, Ningde, Fujian, China; 2 Department of Joint Surgery and Sports Medicine, Ningde Municipal Hospital of Ningde Normal University, Ningde, Fujian, China

**Keywords:** total knee arthroplasty (TKA), intradermal suture, wound healing, incision suture, cosmetic suture, suture technique

## Abstract

**Background:**

In recent years, intradermal suture has gained increasing popularity due to its aesthetic incision and minimize scar formation. However, its efficacy compared to conventional intermittent suture in total knee arthroplasty (TKA) remains unclear. Therefore, we performed a retrospective study to evaluate the association between these two skin closure techniques and outcomes in patients underwent TKA.

**Methods:**

A total of 100 patients with unilateral knee osteoarthritis, who underwent TKA between October 2020 and October 2022 in our hospital, were selected based on inclusion and exclusion criteria. Patients were allocated to either a traditional suture group or a modified suture group according to skin closure method, with 50 patients in each group. The cohort consisted of 44 males and 56 females, with a mean age of 67.50 ± 6.14 years (range 57–79) and a mean disease duration of 8.26 ± 4.05 years (range 1–19). Hollander Wound Evaluation Score (HWES), Patient Scar Assessment Score (PSAS), and Observer Scar Assessment Score (OSAS) were compared 1,2,6,12,24 weeks postoperative, while the Range of Motion (ROM) were assessed 6,12,24 weeks post-surgery and Visual Analog Scale (VAS) were compared 1,3,5,7,14 days postoperative. Data collection was performed by independent assessors.

**Results:**

Compared to the traditional group, the incision suture time, number of suture reactions, postoperative hospitalization time, PSAS, and OSAS of the modified group were lower. The VAS scores of the modified group were lower than those of the traditional group at 3, 5, and 7 days postoperative; the HWES scores at 1, 2, 6, 12, and 24 weeks postoperative; and the satisfaction scores of the incision aesthetics were significantly greater than those of the traditional group (*P* < 0.05). The number of incision dressing changes, incision alignment cases, incision exudation cases, and non-grade A healing cases in the modified group were slightly lower than those in the traditional group, with no statistical significance (*P* > 0.05). The flexion angle, extension angle, and flexion-extension angle of the knee joints in the two groups at 6, 12, and 24 weeks postoperative were significantly greater than those at baseline (*P* < 0.05), but the differences were not statistically significant (*P* > 0.05).

**Conclusion:**

The modified intradermal suturing technique was associated with significant improvements in scar condition and patient satisfaction with the aesthetic outcome of the incision. It was associated with shorter suturing time, lower incidence of suture reactions, and shorter postoperative hospitalization time compared with the traditional intermittent method.

## 1 Introduction

Knee osteoarthritis (KOA) is a common degenerative disease of the knee joint in middle-aged and elderly people that severely affects the joint function and quality of life of patients. Total knee arthroplasty (TKA) is a mature and effective treatment for end-stage KOA, that improves patients' quality of life by relieving pain and restoring joint function (1–3). With the intensification of population aging, the number of patients with KOA is increasing annually, and the number of patients receiving TKA is also growing annually. The number of annual TKAs in the United States is expected to reach 3.5 million by 2030 (4). With the promotion and popularization of TKA technology, as well as the implementation of the enhanced recovery after surgery (ERAS) concept—a multimodal perioperative care pathway or protocol designed to facilitate early recovery and shorten hospital stays for patients undergoing major surgery, which is patient-centered, multimodal, multidisciplinary, and evidence-based—patients and clinicians have raised higher demands for TKA technology (5).

At present, the dissatisfaction rate of TKA is still as high as 20%, and the reasons include prosthesis loosening, periprosthesis infection, pain, and incision-related complications (6). The use of incision sutures has received increasing attention to improve patient satisfaction, functional scores, and rapid recovery after joint arthroplasty. At present, there are many incision suture methods for TKA (7–10), such as traditional intermittent suture, subcutaneous continuous suture, and skin stapler suture. However, in practice, the selection of suture methods is based mainly on the clinical experience of doctors, and there is no unified standard.

Traditional intermittent suture incisions involve a “centipede leg” scar, which severely affects aesthetics. In clinical practice, patients often require cosmetic sutures. Moreover, according to clinical observation traditional intermittent suturing via mousse thread (a commonly used medical suture in clinical) requires suture removal after surgery, which increases the hospitalization time for some patients. With increasing use of the ERAS concept, clinicians are becoming more willing to use intradermal suturing, which helps reduce hospitalization time and improves patient satisfaction. Therefore, we selected 100 patients with KOA who underwent TKA from October 2020 to October 2022 in our hospital as the study subjects. The modified intradermal suture method and traditional intermittent suture method were used to close the incision, and the clinical effects of the two groups of patients were observed, as reported below.

## 2 Materials and methods

### 2.1 Patient selection

This retrospective study analyzed 100 patients with unilateral KOA who underwent TKA at our hospital between October 2020 and October 2022 in our hospital, were selected based on inclusion and exclusion criteria. Informed consent was obtained from all participants, and the study received approval from the Ethics Committee (Number: NSYKYLL-2025-60). The report adheres to the STROBE (Strengthening the Reporting of Observational Studies in Epidemiology) guidelines.

Inclusion criteria: (1) Primary unilateral total knee arthroplasty; (2) Patient age <80 years; (3) Kellgren-Lawrence grade (11) of osteoarthritis III-IV; (4) body mass index (BMI) ≤40 kg/m^2^ (12); (5) the surgeries were performed by the same group of doctors according to a unified standard; (6) postoperative rehabilitation training under the guidance of the same rehabilitators; and (7) the follow-up period was ≥6 months for those with complete follow-up data.

**Figure 1:**
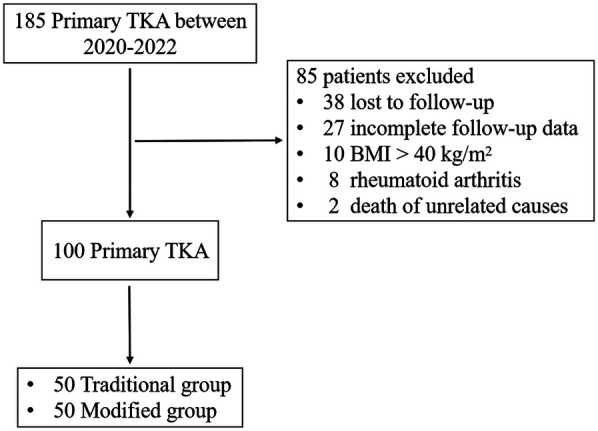
Flowchart of patient selection in the study.

Exclusion criteria: (1) poor skin and soft tissue conditions in the surgical area, such as old scars and sinus tracts; (2) severe malnutrition (lbumin <22 g/L) or consumptive diseases, such as tuberculosis, hyperthyroidism and tumors; (3) long-term smoking history(10 pack-years); (4) long-term use of hormones (>3 months), immunosuppressants (>6 months), and other drugs; and (5) postoperative follow-up time of less than 6 months. The sample selection of the study is shown in Figure 1.

### 2.2 General information

A total of 100 cases were included, including 44 males and 56 females, aged between 57 and 79 years, with an average age of (67.50 ± 6.14) years, and the course of disease ranged from 1 to 19 years, with an average course of (8.26 ± 4.05) years. All patients with unilateral KOA (Kellgren-Lawrence grade III-IV) and were divided into the traditional group and the modified group according to skin closure method, with 50 patients in each group. The traditional intermittent suture method was used to close the incision as in the traditional group, and the modified intradermal suture method was used to close the incision as the modified group. There were no statistically significant differences in general information such as age, gender, side, BMI, disease duration, and grade of osteoarthritis between the two groups, indicating comparability (*P* > 0.05, Table 1). All patients were treated with posterior stabilizing knee prosthesis (PS prosthesis, Zimmer&Biomet). The Kellgren-Lawrence grading of KOA was performed by two highly trained and clinically experienced orthopedic physicians who read x-ray films and conducted consistency tests. After strict inter group and intra group consistency correction, the Kappa coefficient between the two measurers was 0.86 (95% CI: 0.77–0.94), and the Kappa coefficient within the measurer was 0.93 (95% CI: 0.83–1.00), indicating the reliability of the measurement results. Similarly, consistency evaluations have also been conducted for other indicators, such as HWES, ROM, OSAS.

**Table 1:** Comparison of general characteristics between the two groups of patients.

Variables	Traditional group	Modified group	Statistical value	*P-*value
Age (year)	67.68 ± 6.33 (57, 79)	67.32 ± 6.01 (57, 79)	0.292^a^	0.771
Gender (M/F)	21/29	23/27	0.162^b^	0.687
BMI (kg/m^2^)	28.67 ± 3.37 (23.4, 34.6)	29.40 ± 3.18 (23.5, 34.7)	−1.121^a^	0.265
Laterality (Left/Right)	25/25	24/26	0.040^b^	0.841
Disease Course (year)	8.44 ± 4.06 (1, 18)	8.08 ± 4.07 (1, 19)	0.443^a^	0.659
K-L Grade (III/IV)	11/39	8/42	0.585^b^	0.444
ASA Grade (Ⅰ/Ⅱ/Ⅲ)	20/26/4	18/27/5	0.294^c^	0.914
Prosthesis (Z/B)	28/22	24/26	0.641^b^	0.423

Values given as mean ± SD or *N*.

BMI, body mass index; K-L, Kellgren-Lawrence; ASA, American Society of Anesthesiologists.

a Independent sample *t*-test.

b *Chi*-square tests.

c Fisher's exact test.

### 2.3 Surgical methods

All patients were treated by the same surgical team, under general anesthesia with a tourniquet (pressure: 40kPa, duration: 60 min) and the medial parapatellar approach (approximately 15 cm in length) was used to expose the joint. The joint capsule, subcutaneous and skin sutures were all performed by the same senior attending physician. No patients underwent patella resurfacing. After the prosthesis was placed, the joint capsule, muscle, and deep fascia were sutured with #1 coated Vicryl absorbable suture (ETHICON), and the subcutaneous tissue was sutured with 2-0 coated Vicryl absorbable suture (ETHICON).

In the modified intradermal suture group, a 4-0 coated Vicryl taper needle absorbable suture (ETHICON) was used at one end of the incision through the subcutaneous layer into the dermis, and then through the subcutaneous layer from the opposite dermis, and the subcutaneous layer was knotted and fixed as a starting needle. Afterward, close to the skin within the dermis layer, the needles were sutured continuously in an S-shaped manner, with each needle entry point corresponding to the opposite anterior needle exit point, and the needles were slightly moved backward, with a needle spacing of approximately 8 mm. After suturing to the other end of the incision, the skin was sutured through the end of the incision, and then the needle was inserted into the skin again through the exit point. The skin was sewn out at intervals of 8 mm, and three needles were sutured in an N-shape to fix the suture. The tail line was cut tightly against the skin.

**Figure 2:**
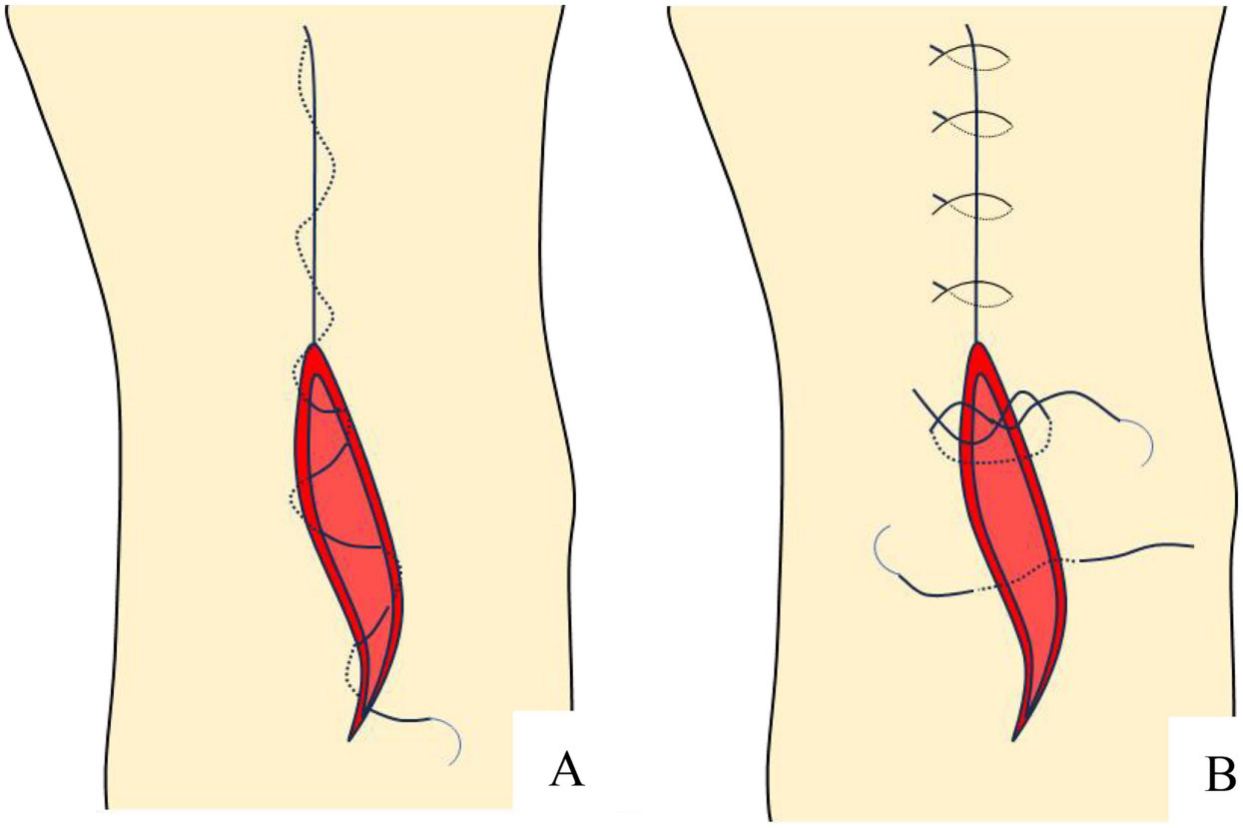
Schematic diagram of incision suture methods. **(A)** Continuous intradermal suture; **(B)** Traditional intermittent suture.

**Figure 3:**
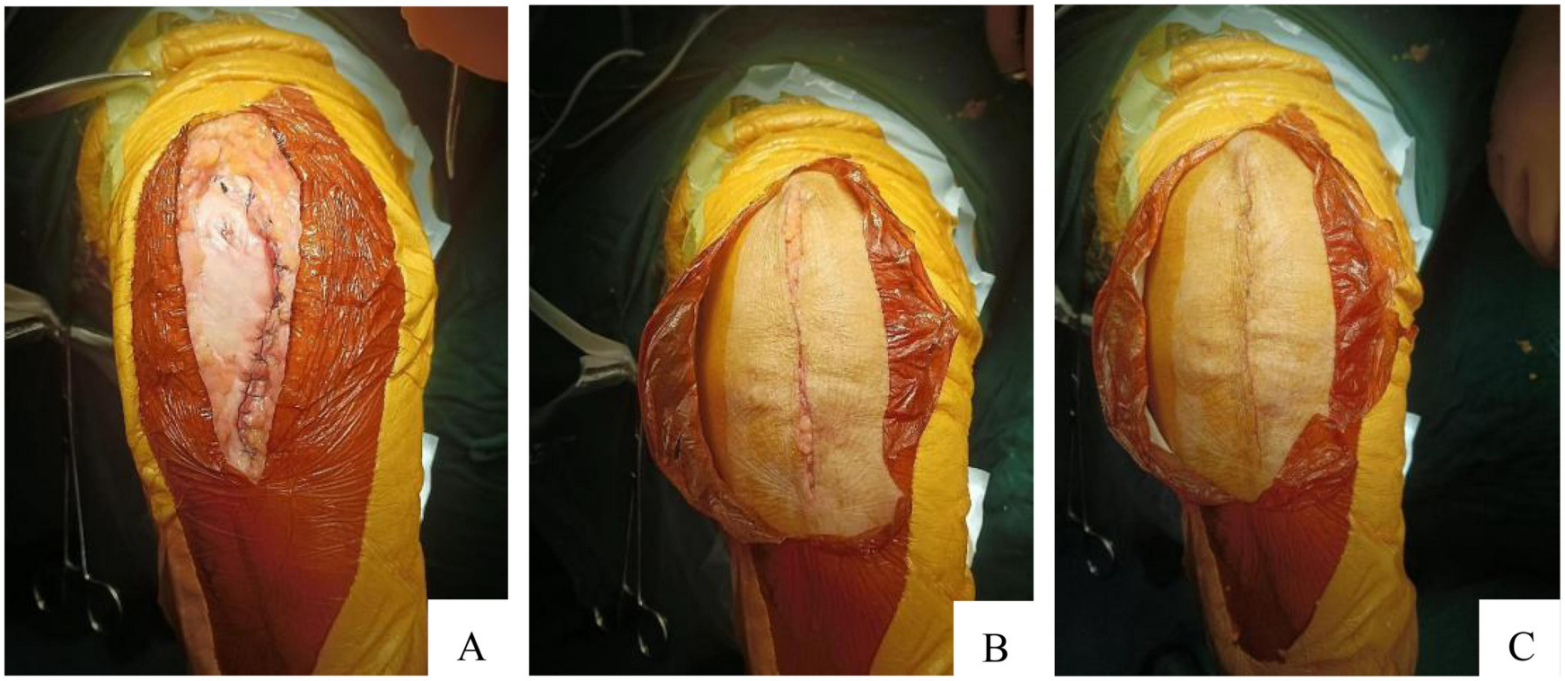
Three layers were sutured. **(A)** Tendon layer closed by absorbable suture; **(B)** Subcutaneous layer closed by absorbable suture; **(C)** Skin layer closed by absorbable suture.

**Figure 4:**
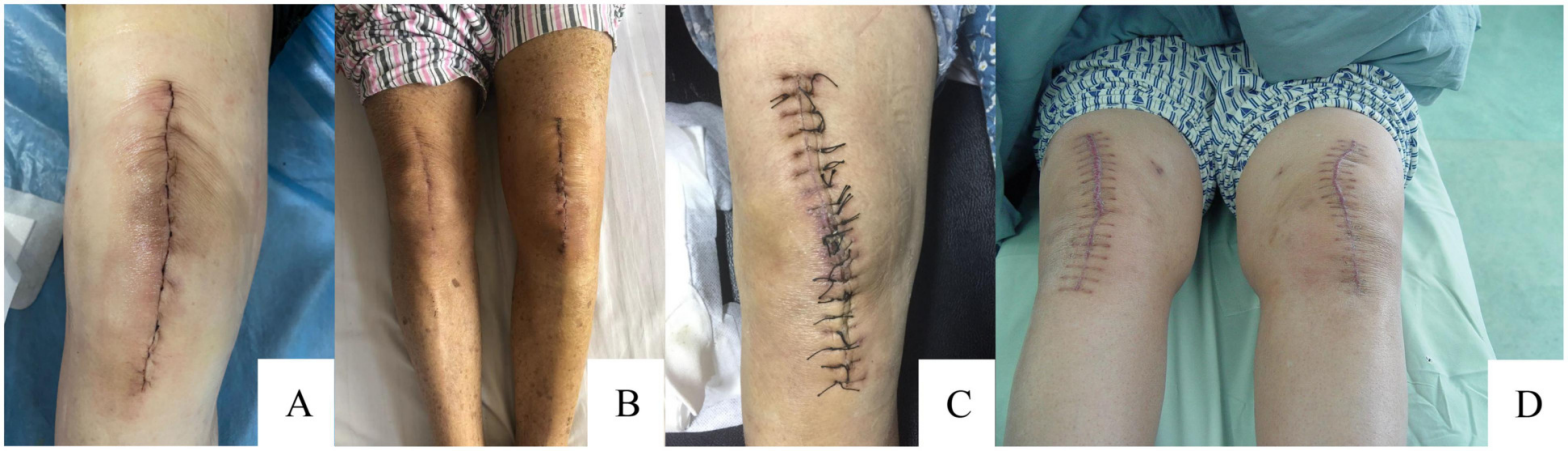
Overview of incision suturing and healing. **(A)** Continuous intradermal suture situation; **(B)** Continuous intradermal suture healing situation; **(C)** Traditional intermittent suture situation; **(D)** Traditional intermittent suture healing situation.

In the traditional intermittent suture group, 4-0 mousse thread triangular needle non-absorbable suture was used to intermittently suture the incision, with a needle spacing of approximately 8 mm and a needle eye distance of approximately 5 mm from the incision. The differences between these two methods are shown in the a diagram in Figure 2. The intraoperative and postoperative incision conditions of the two groups are shown in Figures 3, 4.

### 2.4 Postoperative management

The postoperative treatment methods of the two groups were consistent. Routine use of antibiotics within 24 h postoperatively to prevent infection, and oral administration of rivaroxaban (1 tablet/day) for 4 weeks postoperatively to prevent deep vein thrombosis in both lower limbs. Remove the drainage tube promptly based on the patient's condition and drainage volume (usually within 48 h postoperatively). Postoperatively, the wounds were covered with a hemostatic dressing applied in the operating room. The surgical limb was wrapped with a thick cotton pad and then compressed with an elastic bandage (from the ankle to 15 cm proximal to the knee joint). The dressing was changed on the second day after surgery and every 2–3 days once depending on the incision condition. All patients underwent compression bandage removal on the second day after surgery and began active and passive knee flexion and extension exercises. In the traditional intermittent suture group, the sutures were removed 12–14 days after surgery depending on the degree of wound healing. Postoperative rehabilitation training under the guidance of the same rehabilitators.

On the first day after surgery, the in-hospital physical therapy plan centered on encouraging early patient mobilization. Patients were guided to bear weight or partially bear weight according to their tolerance levels. The rehabilitation guidelines were intended to help patients regain the ability to perform daily activities independently. This was achieved through a structured program that included exercises done in bed, range of motion (ROM) exercises, activities to strengthen the lower limbs, training on how to walk properly, and practice sessions for climbing stairs.

The data collection was handled by trained data administrators responsible for data entry and management, using a dual entry verification mechanism to ensure the accuracy and completeness of the data.

### 2.5 Observation indicators

#### 2.5.1 Main outcome measures

Hollander wound evaluation score (HWES) (13), patient scar assessment score (PSAS) (14), observer scar assessment score (OSAS) (15), incision aesthetic satisfaction score.

#### 2.5.2 Secondary outcome measures

Incision suture time (referring only to skin suture time), dressing changes frequency, incision complications (redness, exudation, dehiscence, subcutaneous hematoma, infection, etc.), evaluation criteria for incision healing, visual analogue scale (VAS) for incision pain (16), range of motion (ROM) of the knee joint, Lysholm score (17) and postoperative hospitalization time.

#### 2.5.3 Postoperative incision pain

The VAS score was used to evaluate the degree of pain at 1, 3, 5,7, and 14 days after surgery. The total score ranges from 0 to 10 points, with 0 points indicating painless and 10 points indicating severe pain. <3 points: Mild pain, tolerable; 4–6 points: Moderate pain, affecting sleep, still tolerable; 7–10 points, severe pain, unbearable, affecting sleep and appetite.

#### 2.5.4 Evaluation criteria for incision healing

In this study, incision healing was defined as follows: the wound edge was close to closure without a cavity, there was no separation of the incision edge, and the tissue structure and function were well repaired. The healing level of the incision was represented by A, B, and C. Grade A healing refers to excellent healing without any adverse reactions; Grade B healing refers to poor healing with inflammatory reactions at the healing site, such as redness, swelling, induration, hematoma, effusion, etc., but without suppuration; Grade C healing refers to wound infection and suppuration, requiring debridement or incision drainage (18). Non -Grade A healing includes Grade B and Grade C healing.

#### 2.5.5 Incision healing status

The HWES was used to evaluate the incision healing status at 1, 2, 6, 12, and 24 weeks after surgery. This scale includes items such as misalignment of incisions, overall aesthetics, misalignment of incisions, inversion of incision edges, excessive distortion, and alignment margins exceeding 2 mm. Each item is worth 1 point, and the total score ranges from 0 to 6 points, with 6 points being the best.

#### 2.5.6 Scar situation

The patient observer scar assessment score (POSAS), including the patient scar assessment score (PSAS) and observer scar assessment score (OSAS) was used to evaluate the degree of the incision scar situation at 1, 2, 6, 12, and 24 weeks after surgery. Among them, PSAS includes 6 items, the score range for each item is 0–10 points, OSAS includes 5 entries, the score range for each item is 0–10 points. The lower the rating, the better the aesthetics.

#### 2.5.7 Incision aesthetic satisfaction

A 5-level scoring system (19) was used, which includes very satisfied (5 points), satisfied (4 points), general (3 points), dissatisfied (2 points), and very dissatisfied (1 point).

#### 2.5.8 Knee function

The Lysholm score, a widely used assessment tool for knee joint function, evaluates eight domains: limping (5 points), support (5 points), locking (15 points), instability (25 points), pain (25 points), swelling (10 points), climbing stairs (10 points) and squatting (5 points). The total score on this scale ranges from 0 to 100, with higher scores denoting greater knee function.

### 2.6 Statistical analysis

The analysis was performed via the SPSS 26.0 statistical software (IBM Corp., Armonk, New York, USA). Quantitative data with normal distribution were presented as mean ± SD (standard deviation). The inter-groups were compared via Two-tailed Student *t*-tests, the intra-group was compared via a one-way repeated-measures analysis of variance (ANOVA), Bonferroni correction was applied to adjust the significance level for multiple comparisons, and the overall effect between groups was analyzed using two-way repeated-measures ANOVA, If Mauchly's test of sphericity was not satisfied, the Greenhouse-Geisser method was used for correction. The count data were presented as the number of cases (n) and rate (%), and the comparison of rates between groups were conducted via the *Chi*-square test or Fisher's exact test. *P*-values <0.05 were considered statistically significant.

## 3 Results

### 3.1 Comparison of the HWES, PSAS, and OSAS score between groups

**Figure 5:**
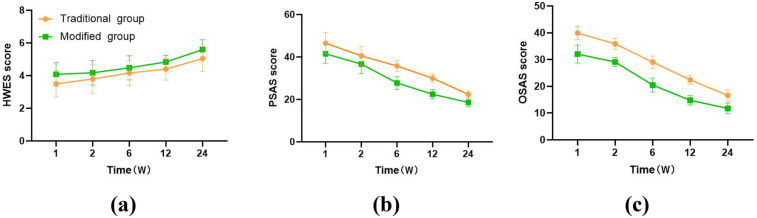
Comparison of the HWES **(a)**, PSAS **(b)**, and OSAS **(c)** score between groups.

HWES scores in the modified group were significantly greater than those in the traditional group at 1, 2, 6, 12, and 24 weeks after surgery (4.08 ± 0.72 vs. 3.50 ± 0.81, 4.18 ± 0.75 vs. 3.80 ± 0.88, 4.48 ± 0.74 vs. 4.16 ± 0.77, 4.84 ± 0.42 vs. 4.40 ± 0.67, 5.60 ± 0.61 vs. 5.04 ± 0.78, all *P* < 0.05, Figure 5a), whereas PSAS and OSAS scores were significantly lower than those in the traditional group (41.52 ± 4.46 vs. 46.54 ± 5.02, 36.70 ± 4.59 vs. 40.56 ± 4.38, 27.76 ± 3.02 vs. 35.78 ± 2.50, 22.48 ± 2.16 vs. 30.02 ± 1.92, 18.62 ± 2.08 vs. 22.38 ± 1.70 and 32.06 ± 3.33 vs. 39.90 ± 2.48, 29.12 ± 1.64 vs. 35.88 ± 2.00, 20.46 ± 2.56 vs. 29.08 ± 2.25, 14.82 ± 1.80 vs. 22.44 ± 1.67, 11.78 ± 1.97 vs. 16.60 ± 2.12, all *P* < 0.05, Figures 5b,c). There were statistically significant differences in the HWES, PSAS, and OSAS scores at different time points between the groups (*P* < 0.001, Figure 5). Over time, the HWES scores of both groups of patients gradually increased, indicating better wound healing; the scores of PSAS and OSAS gradually decreased, indicating a better aesthetic appearance of the incision.

### 3.2 Comparison of incision observation indicators between groups

The suture time in the modified intradermal suture group was significantly shorter than that in the traditional intermittent suture group, and the number of postoperative suture reactions was significantly lower than that in the traditional intermittent suture group, with statistical significance (*P* < 0.05, Table 2). The number of postoperative dressing changes, cases of poor incision alignment, cases of incision exudation, and cases of non-grade A healing in the modified intradermal suture group were all significantly lower than those in the traditional intermittent suture group, with no statistical significance (*P* > 0.05, Table 2). The postoperative hospitalization time of the modified intradermal suture group was shorter than that of the traditional intermittent suture group, whereas the satisfaction score for incision aesthetics was significantly greater than that of the traditional intermittent suture group, with statistical significance (*P* < 0.05, Table 2).

**Table 2:** Comparison of observation indicators between groups.

Variables	Traditional group	Modified group	Statistical value	*P-*value
Suture time (min)	6.34 ± 1.17 (4, 8)	3.62 ± 0.75 (3, 5)	13.813^a^	<0.001
Dressing changes	3.24 ± 0.66 (2, 4)	3.08 ± 0.78 (2, 4)	1.111^a^	0.269
Poor incision alignment (%)	10 (20)	5 (10)	1.961^b^	0.161
Suture reaction (%)	17 (34)	6 (12)	6.832^b^	0.009
Incision exudation (%)	12 (24)	8 (16)	1.000^b^	0.317
Non-grade A healing (%)	7 (14)	4 (8)	0.919^b^	0.338
Postoperative hospitalization time (day)	10.78 ± 3.28 (7, 15)	6.44 ± 1.16 (5, 9)	8.822^a^	<0.001
Incision aesthetics satisfaction scores	2.96 ± 0.73 (2, 4)	4.60 ± 0.64 (3, 5)	11.979^a^	<0.001

Values given as mean ± SD or *N*.

a Independent samples *t*-test.

b *Chi*-square test.

### 3.3 Comparison of the VAS score between groups

**Figure 6:**
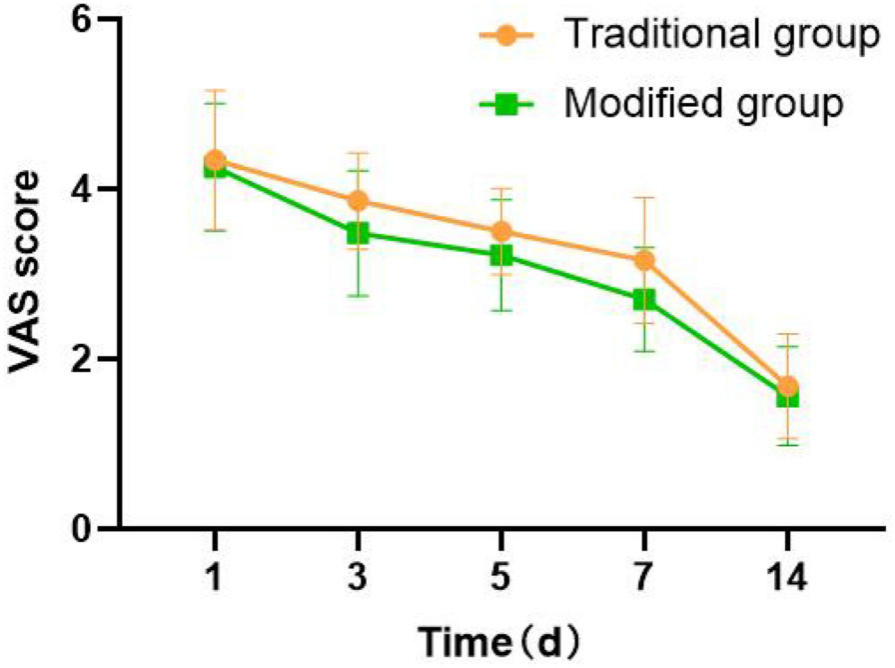
Comparison of the VAS score between groups.

There was no statistically significant difference in the VAS score at 1 and 14 days after surgery between the two groups (4.34 ± 0.82 vs. 4.26 ± 0.75 and 1.68 ± 0.62 vs. 1.56 ± 0.58, both *P* > 0.05, Figure 6). The VAS score of the incision in the modified group was significantly lower than that in the traditional group at 3, 5, and 7 days after surgery, with statistical significance (3.86 ± 0.57 vs. 3.48 ± 0.74, 3.50 ± 0.51 vs. 3.22 ± 0.65, 3.16 ± 0.74 vs. 2.70 ± 0.61, all *P* < 0.05, Figure 6). There was a statistically significant difference in the VAS score at different time points within the groups (*P* < 0.001, Figure 6). Both groups of patients showed a gradual decrease in VAS scores over time, indicating a gradual relief of pain.

### 3.4 Comparison of the knee joint ROM and Lysholm score between groups before and after surgery

**Figure 7:**
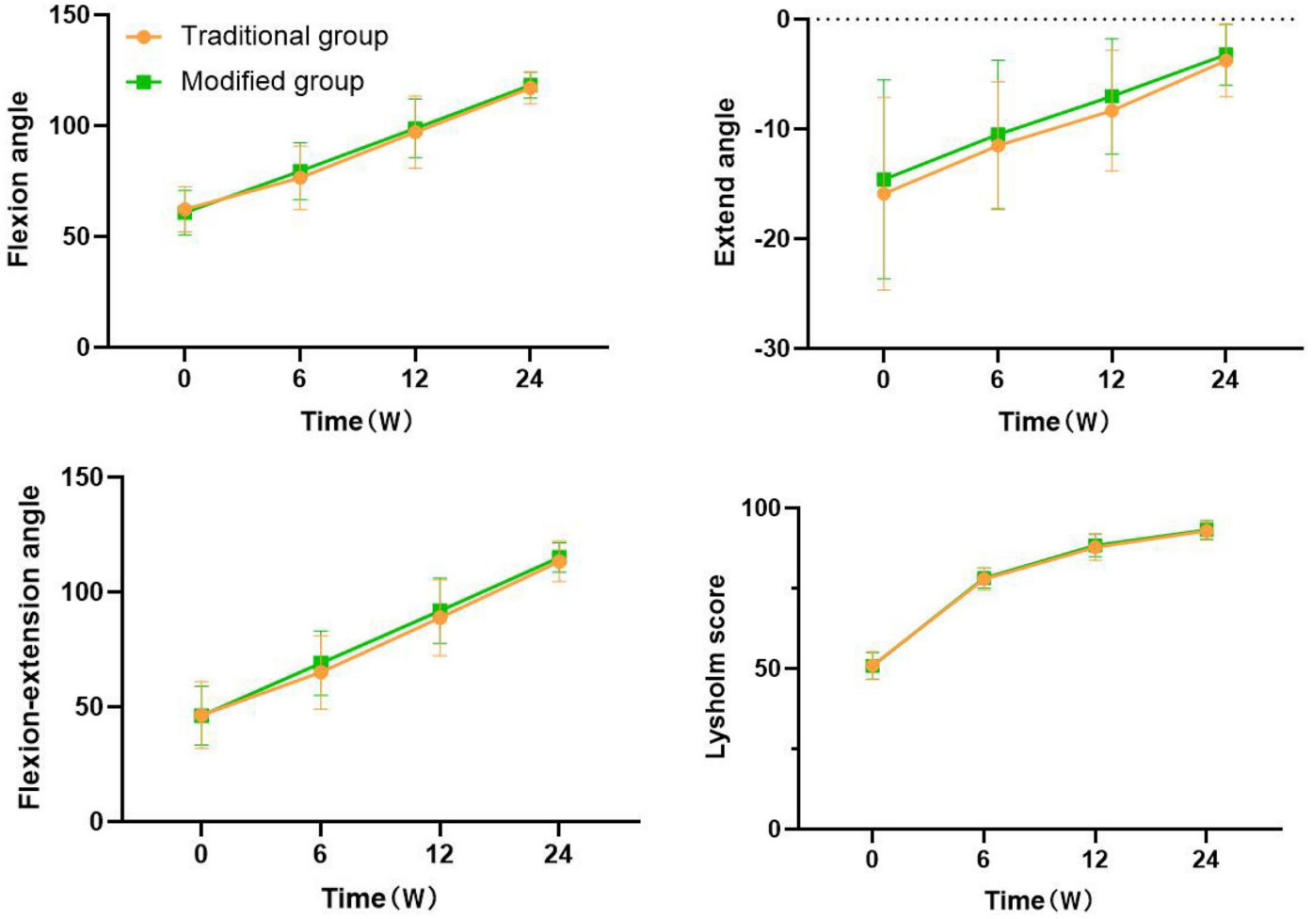
Comparison of the knee joint ROM and lysholm score between groups.

There was no statistically significant difference in the flexion angle, extension angle, flexion-extension angle (flexion and extension angle = flexion angle + extension angle), and Lysholm score of the knee joint between the two groups before surgery (62.36 ± 10.21° vs. 60.78 ± 10.01°, −15.88 ± 8.78° vs. −14.58 ± 9.07°, 46.48 ± 14.46° vs. 46.20 ± 12.73°, and 51.04 ± 4.31 vs. 50.86 ± 4.14, all *P* > 0.05, Figure 7). Compared with those before surgery, the knee flexion angle, extension angle, flexion-extension angle, and Lysholm score of the two groups improved significantly after 6, 12, and 24 weeks of surgery, with statistical significance (Traditional Group: 76.52 ± 14.35° or 97.10 ± 16.28° or 117.12 ± 7.21° vs. 62.36 ± 10.21°, −11.52 ± 5.81° or −8.32 ± 5.50° or −3.76 ± 3.27° vs. −15.88 ± 8.78°, 65.00 ± 15.92° or 88.78 ± 16.63° or 113.36 ± 8.77° vs. 46.48 ± 14.46°, and 77.88 ± 3.45 or 87.80 ± 4.16 or 92.84 ± 2.82 vs. 51.04 ± 4.31; Modified Group: 79.52 ± 12.85° or 98.84 ± 13.19° or 118.28 ± 5.84° vs. 60.78 ± 10.01°, −10.48 ± 6.75° or −7.02 ± 5.26° or −3.22 ± 2.77° vs. −14.58 ± 9.07°, 69.04 ± 13.99° or 91.82 ± 14.23° or 115.06 ± 6.39° vs. 46.20 ± 12.73°, and 78.20 ± 3.19 or 88.40 ± 3.52 or 93.18 ± 2.93 vs. 50.86 ± 4.14; all *P* < 0.001, Figure 7), but there was no statistical significance between the two groups at different time points (*P* > 0.05, Figure 7). As time went on, the flexion andextension angles of the knee joint gradually increase in both groups of patients, indicating that knee joint function was gradually improving.

### 3.5 Comparison of postoperative complications between groups

Modified group: One patient developed exudation at the distal end of the incision 19 days after surgery, which was caused by subcutaneous suture knots leading to point like rupture of the skin incision. The exudation stopped after pressure bandaging; One case had a 1 cm split in the middle of the incision, which improved after pressure bandaging. It is considered that there is a high possibility of low local tension; Two cases showed bloody exudate in the middle of the incision, which improved after pressure bandaging. It is considered that there is a high possibility of local hematoma. Traditional group: 3 cases of continuous exudation at the distal end of the incision, which improved after pressure bandaging; One case of continuous exudation at the distal end of the incision did not show significant improvement after pressure bandaging, so it improved after suturing; One case had a 1 cm split in the middle of the incision, which improved after pressure bandaging, possibly due to a larger suture distance; Two cases of redness and swelling around the incision site gradually disappeared after 14 days of suture removal, indicating a high possibility of suture reaction. There was no statistically significant difference in the incidence of postoperative incision complications between the two groups of patients (χ^2^ = 0.919, *P* = 0.338, Table 3).

**Table 3:** Incision healing outcomes between groups.

Group	*n*	Grade A healing	Grade B healing	Grade C healing
Traditional group	50	43	7	0
Modified group	50	46	4	0

## 4 Discussion

Skin suturing is the final step of knee arthroplasty. Common suture methods include simple intermittent suturing and continuous intradermal suturing. In addition to the different suture methods used, the suture materials used are also differ. The materials currently used include metal nails, sutures, and skin adhesives. Intermittent sutures usually use non-absorbable sutures such as PROLENE, mousse thread, or metal nails, which have the advantages of firm knotting, strong tension resistance, and easy adjustment of tension, and were the most commonly used suture methods in the past. In recent years, some scholars have proposed the method of barbing sutures (20–22). However, its clinical application effect is still controversial (23). Owing to their convenient operation and fast suture speed, skin staples are widely used in surgery. However, owing to the use of metal materials, specialized tools are required to remove the wound after healing, which is more inconvenient, and the cost and incidence of inflammation in the nail eye are greater than those of ordinary mousse thread (24, 25). With improvements in surgical technology and the increasing demand of patients for postoperative incision aesthetics, this method affects incision healing because of the time-wired knot reaction or nail eye inflammation. As non-absorbable sutures or metal materials are needed, sutures or skin nails need to be removed, and a “centipede leg” scar that affects aesthetics after incision healing is inevitable.

A review of the literature indicates that intradermal suture technology has been extensively applied across various surgical disciplines, including plastic surgery (26), neurosurgery (27), gynecology (28), general surgery (29), and orthopedics (30). Continuous intradermal suturing has the advantages of simple operation, fast suturing, minimal damage, excellent skin edge alignment, a beautiful incision, and a small scar, which could improve patient satisfaction and conform to the concept of ERAS (31–33). In clinical practice, absorbable sutures (ETHICON, antimicrobial Vijo) or non-absorbable sutures (PROLENE) are commonly used for intradermal suture, and the use of absorbable suture materials has advantages of no suture removal and reducing the degree of pain associated with suture removal for patients and has been increasingly widely used in clinical practice (34). In this study, the 4-0 coated Vicryl absorbable suture (ETHICON) with good histocompatibility was used for continuous intradermal suture, which has significant advantages over intermittent suture with mousse thread in terms of suture time, suture reaction, and the aesthetics score of the incision. Research has shown that the choice of suture method and suture material has a significant impact on incision healing (23, 35).

In this study, two common methods of skin suturing in TKA were compared and analyzed. In terms of suture time, each stitch of intermittent suture stitch requires knotting and trimming, and the surgical nurse needs to cooperate with threading and needle delivery to prolong the suture time. On the other hand, continuous intradermal suturing only requires knotting and cutting on both sides of the incision, which takes a significantly shorter time than dose intermittent suturing, which is conducive to reducing the risk of postoperative infection. Continuous intradermal suturing is associated with fewer complications than intermittent suturing in terms of poor incision alignment, suture reactions, and incision exudation. The use of continuous intradermal sutures with absorbable thread is associated with fewer overall complications, fewer dressing changes, a higher Grade A healing rate, avoids suture removal, and significantly shorter hospitalization time. Previous studies have shown that the incidence of incision complications after TKA can reach 29%, and once they occur, they will affect the postoperative rehabilitation process, prolong hospitalization time, and reduce surgical outcomes (36). The postoperative incision VAS score of the intradermal suture group was lower than that of the intermittent suture group, indicating a lack of evident dermal puncture sites in the intradermal suture group. Consequently, patients experienced reduced postoperative pain; however, this did not significantly impact the knee joint ROM and Lysholm score in either groups. Compared with intermittent suture group, the intradermal suture group had better scores for incision aesthetic satisfaction, HWES, PSAS, and OSAS compared to the intermittent suture group. This led to improved surgical satisfaction among patients, achieved the goal of skin cosmetic suturing, and accelerated the rehabilitation surgery requirements. Just as mousse threads can provide sufficient strength to the incision, eliminating concerns about incision dehiscence due to knee functional exercises, studies have also shown that absorbable sutures can meet the requirements of knee rehabilitation exercises without causing incision dehiscence.

Overall, the findings substantiate the substantial associations between the modified intradermal suture and better outcomes, particularly in incision healing quality and aesthetic results. These results align with current trends in advancing TKA incision suturing techniques both domestically and internationally (34, 37). Vincent et al. (38) demonstrated that interrupted suturing with non-absorbable sutures has several disadvantages, including increased suture knots, a higher risk of infection, prolonged operation time, longer hospital stays, higher costs, and the formation of unsightly “centipede leg” scars. Zhou et al. (39) further confirmed that, compared to intermittent suturing, intradermal suturing with barbed suture after TKA provides advantages in safety, effectiveness, shorter surgical time, improved cosmetic outcomes, and higher patient satisfaction.

This study still has certain limitations, such as a relatively small sample size, single center, and retrospective case studies. As a retrospective study, there is potential for selection and researcher biases that could affect the generalizability of the findings. The limited sample size and short follow-up period inherent in single-center retrospective studies also mean that the long-term efficacy and broader applicability of this method require further validation through large sample, prospective multicenter randomized controlled trials.

The study mainly focuses on the influence of different skin suturing methods on the healing of TKA surgical incisions, without involving suture materials. Due to the fact that TKA patients are mainly elderly, with low education level, insufficient understanding of rehabilitation knowledge, and poor compliance, the impact on postoperative functional rehabilitation needs further research and clarification. Additionally, the study did not account for confounding factors such as smoking, obesity, and diabetes. Given the high prevalence of these conditions in middle-aged and elderly populations, future studies should incorporate these factors into their design to minimize potential confounding variables, thus enhancing the validity and reliability of the results.

## 5 Conclusion

Compared with the traditional intermittent suture method, the modified intradermal suture method is not associated with changes in postoperative rehabilitation function exercise of the knee joint, and is associated with shorter incision suture time, shorter postoperative hospitalization time, lower suture reactions, alleviated postoperative incision pain, improve scar situation of the incision, and enhance incision aesthetics satisfaction of TKA patients. This technique is more consistent with the concept of ERAS and is an effective suture method.

## Data Availability

The original contributions presented in the study are included in the article/Supplementary Material, further inquiries can be directed to the corresponding authors.
